# Keraunoparalysis: Fleeting Paralysis Following a Lightning Flash

**DOI:** 10.7759/cureus.70773

**Published:** 2024-10-03

**Authors:** Nidhi Elizabeth Jacob, Rashika M

**Affiliations:** 1 Department of Internal Medicine, Government Medical College Mahasamund, Mahasamund, IND; 2 Department of Radiodiagnosis, Government Medical College Mahasamund, Mahasamund, IND

**Keywords:** benign condition, cherrington's classification, keraunoparalysis, lightning strike, transient paralysis

## Abstract

Lightning strikes cause a spectrum of manifestations in the human body. Keraunoparalysis is a neurological condition where immediate but transient weakness occurs following a lightning strike. Herein, we report a case of a 45-year-old male with an acute onset of right-sided hemiparesis after a lightning strike. The patient was investigated for probable causes of paralysis with basic investigations and imaging modalities, which did not reveal any significant findings explaining the symptoms. After eliminating other causes of acute neurological deficit, we suspected it to be a case of keraunoparalysis. The patient was observed closely for further progression of symptoms. The patient was managed conservatively, and analgesics were given to relieve the pain. The symptoms started improving in the first 12 hours, with complete resolution within a week of the impact. This case was reported to increase clinicians' awareness of this benign condition to avoid therapeutic mismanagement. Diagnosing keraunoparalysis is possible only after ruling out the other probable causes of neurological deficits.

## Introduction

Lightning strikes the earth more than 100 times each second, more than eight million times daily. It is the second leading cause of weather-related mortality after death due to extreme temperatures. The clinical manifestations vary from burn injuries to multisystemic involvement with cardiovascular, renal, and neurological abnormalities [1]. Keraunoparalysis is a benign neurological condition that presents as transient limb weakness following a lightning strike. The word keraunoparalysis comes from the Greek root words keraunós, meaning "lightning," and paraluesthai, meaning "be disabled at the site." It is also called lightning paralysis or Charcot paralysis, after the 19th-century French neurologist who first described the condition [2]. Keraunoparalysis may hinder people from leaving unsafe locations, thus further increasing mortality.

## Case presentation

A 45-year-old male was brought to the emergency department of Government Medical College Mahasamund, Kharora, Chhattisgarh, with an alleged history of lightning strikes while working in a paddy field. He was unconscious for five minutes, and after regaining consciousness, he was unable to move his right upper and lower extremities. He also complained of generalized body aches. There was no history of seizures and chronic illnesses like diabetes and hypertension.

The patient was conscious, with a Glasgow Coma Scale of 15/15, and exhibited clear orientation to time, place, and person. Upon examination, he was found to be afebrile, with a heart rate of 80 beats/minute and a blood pressure of 110/80 mmHg. There were no burns or external injuries. During the CNS examination, it was observed that there was right hemiparesis with a power of 3/5 in the upper and lower extremities. Tone and deep tendon reflexes were diminished on the right compared to the left. Plantar reflex showed flexor response bilaterally. There was marked impairment of all modalities of sensation on the affected side. No obvious cranial nerve palsies were noted. All other systems were within normal limits. The patient's full blood count, liver function, and kidney function tests all showed results within the normal range, as detailed in Table 1.

**Table 1:** Blood investigations IU: international units

Parameter	Result	Reference range
Hemoglobin (g/dL)	14	13.5-17.5
Total leucocyte count (per mcL)	5,000	4,500-11,000
Platelet count (per mcL)	180,000	150,000-450,000
Total bilirubin (mg/dL)	0.8	0.1-1.2
Aspartate transaminase (IU/L)	23	<40
Alanine transaminase (IU/L)	22	<40
Serum urea (mg/dL)	9	5-20
Serum creatinine (mg/dL)	0.8	0.6-1.2
Troponin I (ng/mL)	0.01	0-0.04
Serum sodium (mEq/L)	137	135-145
Serum potassium (mEq/L)	4	3.5-5
Prothrombin time (second)	12	11-15
Activated partial thromboplastin time (second)	30	25-40

**Figure 1:**
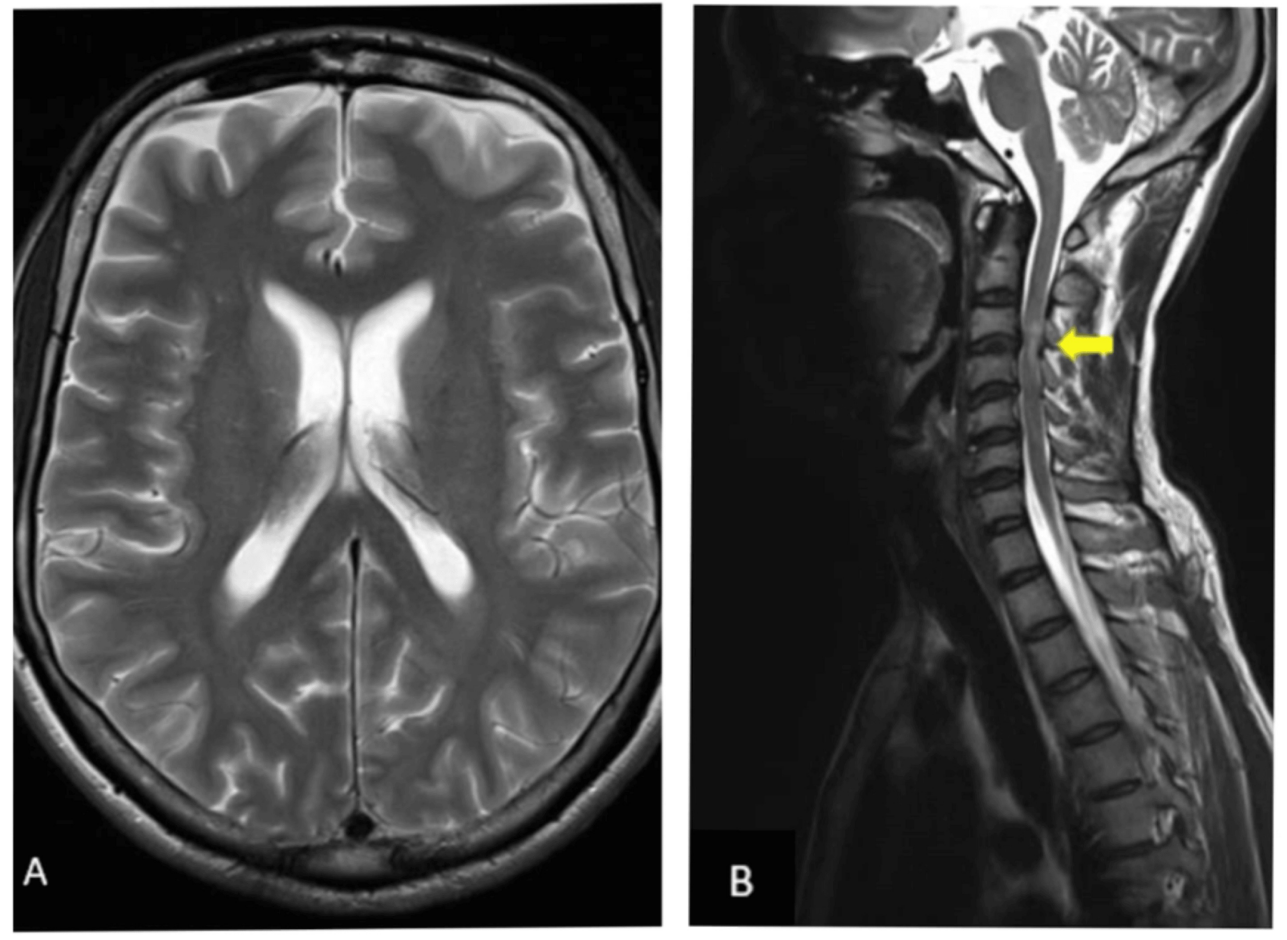
(A) The T2-weighted MRI axial view of the brain showing no abnormality. (B) The T2-weighted MRI sagittal view of the cervical spine showing disc bulge with the altered signal intensity of cord (yellow arrow), suggestive of prolapsed intervertebral disc, a chronic nonspecific finding

The 12-lead electrocardiogram (ECG) revealed no evidence of ischemia. Chest X-ray showed bilateral clear lung fields, normal cardiac shadow, and no obvious fractures. CT head showed no obvious abnormalities in the brain parenchyma. MRI brain (stroke protocol) showed normal brain parenchyma. Additional whole spine screening revealed altered signals in the cord at the C3-C4 vertebral level due to a prolapsed intervertebral disc, which is most probably a nonspecific chronic finding (Figure 1).

The patient was closely monitored over the next few days. The patient received 0.9% normal saline and was given parenteral diclofenac 50 mg every 12 hours along with ranitidine. The symptoms improved within the first 12 hours, with complete recovery of weakness and restoration of sensory deficit noted within a week.

## Discussion

Lightning strikes are categorized as high-voltage injuries, subjecting the body to over 1,000,000 Volts and 10,000-200,000 Amperes of current for a short duration ranging from approximately 1/1,000 to 1/10 seconds. Due to the brief exposure period, victims mainly escape the detrimental effects of the lightning bolt. This leads to only a small amount of energy being internally transmitted, while the majority flows externally over the body. This phenomenon is known as the flashover effect. In addition to electrical injuries, patients can be injured by extreme temperatures or blast waves [3].

The clinical presentation in lightning strike victims varies from individual to individual. Tissue having the least resistance to the flow of charge (nerve < blood < muscle) is most susceptible to injury, making nervous and cardiovascular manifestations common [4]. The neurological manifestation ranges from transient benign symptoms to permanent neurological deficits and is classified according to Cherington's classification (Table 2) [5].

**Table 2:** Cherington's classification

Group	Characterization	Features
1	Immediate and transient	Unconsciousness, amnesia, confusion, keraunoparalysis, weakness, paraesthesia, and headache
2	Immediate and prolonged/permanent	Intracranial hemorrhage, cerebral infarction, post-hypoxic-ischemic encephalopathy, cerebellar syndromes, neurocognitive disorders, and spinal and peripheral nerve injuries
3	Delayed	Neurological syndromes, motor neuron diseases, and movement disorders
4	Lightening-related trauma	Due to falls or blasts such as epidural, subdural, and subarachnoid hemorrhage

Keraunoparalysis is a temporary paralysis affecting one or more limbs after a lightning strike. It spontaneously reverses in a matter of a few hours to days. The clinical symptoms include flaccid paralysis with or without sensory loss. It quite often affects the lower limb compared to the upper limbs [6]. It is presumed to be caused by vasospasm of spinal arteries due to the overstimulation of the autonomic nervous system. It can also present with features of poor peripheral circulation of the affected limbs, such as pale, pulseless, cold, and discolored skin due to vasoconstriction of the extremities [1,7]. In unconscious patients, it mimics cardiac arrest because of the pulseless peripheral limb. Hence, caution should be taken to assess the central pulse before starting the resuscitation. In addition to keraunoparalysis, lightning injury is associated with other types of autonomic nervous system dysfunction, such as complex regional pain syndrome (CRPS) and cardiovascular abnormalities. The manifestation of CRPS is pain, hyperpathia, sweating, and edema [8].

The cardiovascular manifestations include immediate cardiac arrest, hypertension, tachycardia, and nonspecific ECG changes. In rare cases, they may also include myocardial infarction, necrosis, and contusion. The Lichtenberg figure represents the most pathognomonic cutaneous pattern. It is associated with full- or partial-thickness burns. It can also present with fractures and damage to other internal organs, including acute renal failure due to rhabdomyolysis. Auditory findings include tympanic membrane rupture, transient vertigo, and, rarely, sensorineural hearing loss. Immediate retinal and optic nerve damage can also be seen. Cataracts can be a late complication [9].

Besides sudden cardiac arrest, lightning strikes do not usually cause any immediate life-threatening injuries. Therefore, moribund should be prioritized by reverse triaging [10]. All lightning strike victims should be hospitalized irrespective of their symptoms, as their condition can deteriorate. Blood investigations, such as complete blood count, myoglobin, electrolytes, blood urea nitrogen, creatinine, creatine phosphokinase, creatine kinase-MB, coagulation panel, and troponin, should be done in all patients. It is important to look for QT prolongation, T-wave inversion, and ST alteration in ECG and myoglobin in urinalysis. Additional neuroimaging is required in case of any new focal neurological deficits, alteration in mental status, and suspected head/spine injury [11]. Vital monitoring is adequate for asymptomatic patients with normal investigations. In contrast, high-risk patients, such as those suspected of direct strike injury, patients presenting with unconsciousness, chest pain, dyspnea, cranial or leg burns, burns >10% of total body surface area, persisting neurological damage, and pregnancy, necessitate intensive care treatment [3].

## Conclusions

Although lightning is one of the most common natural phenomena, examining the current literature reveals a paucity of cases of keraunoparalysis. It is highly possible that cases are underreported. Nevertheless, properly understanding this condition is imperative for prompt analysis and management of any such patient. Despite many recommendations to avoid unnecessary investigations in suspected patients, it will be prudent to rule out any other causes of neurological deficits before the diagnosis of keraunoparalysis can be considered. It is important to appreciate the benignity of this condition, with complete resolution of symptoms within a short duration. This will help ease unwanted stress and keep fear at bay.
